# A Flexible Sensing Material with High Force and Thermal Sensitivity Based on GaInSn in Capillary Embedded in PDMS

**DOI:** 10.3390/polym16233426

**Published:** 2024-12-05

**Authors:** Fandou Bao, Fengyao Ni, Qianqian Zhai, Zhizhuang Sun, Xiaolin Song, Yu Lin

**Affiliations:** 1 School of Physics and Electronic Engineering, Jining University, Qufu 273155, China; baofandou@163.com (F.B.);; 2 Shandong Donghong Pipe Industry Co., Ltd., Qufu 273155, China

**Keywords:** flexible sensing material, force sensitivity, thermal sensitivity, capillary GaInSn, PDMS

## Abstract

Flexible sensing materials have become a hot topic due to their sensitive electrical response to external force or temperature and their promising applications in flexible wear and human–machine interaction. In this study, a PDMS/capillary GaInSn flexible sensing material with high force and thermal sensitivity was prepared utilizing liquid metal (LM, GaInSn), flexible silicone capillary, and polydimethylsiloxane (PDMS). The resistance (*R*) of the flexible sensing materials under the action of different forces and temperatures was recorded in real-time. The electrical performance results confirmed that the *R* of the sensing material was responsive to temperature changes and increased with the increasing temperature, indicating its ability to transmit temperature signals into electrical signals. The *R* was also sensitive to the external force, such as cyclic stretching, cyclic compression, cyclic bending, impact and rolling. The Δ*R*/*R*_0_ changed periodically and stably with the cyclic stretching, cyclic compression and cyclic bending when the conductive pathway diameter was 0.5–1.0 mm, the cyclic tensile strain ≤ 20%, the cyclic tensile rate ≤ 2.0 mm/min, the compression ratio ≤ 0.5, and the relative bending curvature ≤ 0.16. Moreover, the material exhibited sensitivity in detecting biological signals, such as the joint movements of the finger, wrist, elbow and the stand up-crouch motion. In conclusion, this work provides a method for preparing a sensing material with the capillary structure, which was confirmed to be sensitive to force and heat, and it produced different types of *R* signals under different deformations and different temperatures.

## 1. Introduction

Flexible electronics technology is a technique of constructing electronic devices on flexible substrates and forming circuits, ensuring that their conductive performance remains unaffected under deformation conditions or changes regularly with deformations. This technology holds tremendous potential for applications in pressure and strain sensors, flexible and stretchable electrodes, energy storage, electronic medicine, human–machine interaction and wearable devices [1,2,3,4,5,6]. In particular, flexible wearable devices have garnered widespread attention for their ability to convert external stimuli such as pressure and temperature into electrical signals, making them valuable in sensors and electronic skin applications [7]. The “flexible substrate + electronic device” approach endows electronic devices with a certain capacity to withstand mechanical deformation. However, electronic devices themselves typically exhibit rigid characteristics and cannot achieve complete flexibility. Furthermore, their integration degree may be limited. Therefore, the development of high-performance flexible conductive materials forms the foundation for achieving intelligent, flexible, and highly integrated electronic devices. The combination of the polymer materials with the conductive media offers not only the unique processability and mechanical properties of the polymers but also the conductive and heat dissipation properties of the conductive media [8,9,10].

Poly(dimethylsiloxane) (PDMS) is an ideal candidate for the elastic substrates of stretchable electronics due to its excellent stretchability, excellent resistance to high and low temperatures, air permeability, good chemical stability, low surface tension, elastic modulus close to human skin [11], and excellent drug release property [12], which makes it an ideal substrate material for the preparation of the flexible wearable devices [13,14].

The combination of PDMS and various conductive fillers for the fabrication of flexible sensing materials paves a new pathway for the development of flexible electronics. For instance, as mentioned in the report of Zhe Liu [15], there have been previous research attempts to fabricate composite sensing materials by constructing conductive fluid channels on PDMS substrates. Similar studies are now being applied in various fields such as flexible sensors and wearable supercapacitors [16]. A number of recent studies have used conductive fillers such as silver nanowires [17], carbon nanotubes [18], silver nanoparticles [19], carbon [20], Cu@TiO_2_ [21], etc., to build conductive networks in PDMS, and adopted reasonable signal channel design methods and controllable manufacturing schemes to improve the mechanical stability of the sensing materials. Moreover, incorporating conductive particles or fiber structures into PDMS to create conductive/sensing channels allows for high conductivity and excellent sensing performance at lower concentrations of conductive fillers. To further enhance the conductive stability of the sensing material, Jianwen Chen [22] developed a stretchable transparent pressure sensor based on a sandwich-like composite of polydimethylsiloxane (PDMS)/carbon nanotubes (CNTs)/PDMS. This approach improved the electrical stability of the material, resulting in strain sensors with excellent stretchability, good optical transparency and sensing performance. Moreover, a stretchable structure design of the sensing material was also proposed, which can achieve stable conductive performance under large deformation [23].

In recent research, liquid metals (LMs) have been receiving more and more attention. LM is a kind of metal or alloy with a low melting point, which can form liquid eutectic at room temperature. It has the properties of both fluid and metal and is the best combination of deformability and electrical conductivity among the existing materials [6,24]. As a result, LM-PDMS composite materials have become a hot topic in sensor fabrication [11,25,26,27,28,29,30,31,32,33]. For example, Jinbin Yang [34] designed a method to pattern LM on a flexible PDMS substrate, which can be used for flexible electronic circuits. This method demonstrates good capability in laying down conductive patterns, which is promising for the development of increasingly complex flexible electronic circuits [34,35]. Jianyu Xu [36] developed a recyclable conductive ink using polyvinyl alcohol and LM, which can be printed on flexible materials to create flexible sensors for monitoring human motion as electronic skin. Additionally, Yuji Gao’s team [37] fabricated a microfiber sensor using PDMS and LM, exhibiting flexibility and conductivity. All these studies have improved the methods of constructing conductive channels to fabricate flexible sensing materials to some extent.

This study developed a fabrication process for a flexible capillary structure sensing material with high stretchability, bendability, and excellent sensing performance through dual encapsulation layers. The manufacturing and usage processes are simple, low-cost, non-toxic, and environmentally friendly, offering significant economic benefits. It provides a new avenue for the development of flexible sensors. The force-sensitive, temperature-sensitive and human movement sensors based on this material as the core were also explored, which will have a certain guiding significance for the development of new flexible sensor materials and their potential applications.

## 2. Materials and Methods

### 2.1. Materials

Silicone capillary (0.5/0.8/1.0 mm) and fine iron wire (1.0 mm), were obtained from Taizhou Chunshi New Material Co., Ltd. (Taizhou, China). The gallium-indium-tin alloy (GaInSn, 99.999%, melting point 11 °C), was obtained from Dongguan Dingguan Metal Technology Co., Ltd. (Dongguan, China). PDMS (SYLGARD^TM^ 184 Silicone Elastomer Kit: Part A is mainly polydimethylmethylvinylsiloxane with tiny amounts of platinum catalysts; Part B is mainly the crosslinking agent polydimethyl-methylhydrogensiloxane; A and B are used with a mass ratio of 10:1), was purchased from Dow Silicones Corporation (Auburn, MI, USA). All the reagents above were used without additional treatment.

### 2.2. Preparation of the PDMS/Capillary GaInSn Flexible Sensing Material

**Figure 1:**
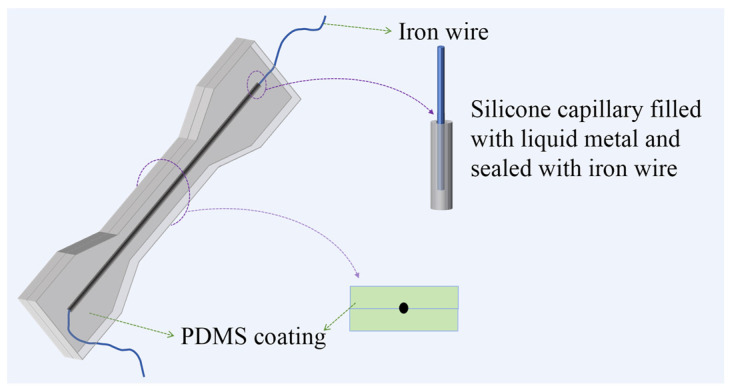
Model diagram of the PDMS/capillary GaInSn flexible sensing material.

First, the LM GaInSn was injected into a 45 mm-long silicone capillary (0.5 mm, 0.8 mm, 1.0 mm). Two 20 mm-long fine iron wires were inserted into both ends of the silicone capillary containing GaInSn for sealing. The sealing operation could prevent the leakage of the GaInSn, which could lead to the circuit interruption before being encapsulated in the PDMS. Moreover, the other end of the wires could be connected to the external circuit after the sample preparation. Second, a certain amount of viscous PDMS prepolymer (Part A:Part B = 10:1) was poured (the thickness of PDMS was about 0.6 mm) into the dumbbell groove with depths of 2.0 mm and cured at 100 °C for 0.5 h. Third, the sealed GaInSn-containing silicone capillary was pressed onto the surface of the first PDMS layer, then the PDMS prepolymer (Part A:Part B = 10:1) was poured into the dumbbell groove until it was filled up, and then was also cured at 100 °C for 0.5 h. Finally, the PDMS/capillary GaInSn material was emoulded. The capillary GaInSn was embedded in the PDMS with the terminal of the fine iron wire exposed, as a result, the PDMS/capillary GaInSn material could be tested without being pierced. The resulting samples are shown in Figure 1.

For comparison, the PDMS + PDMS/GaInSn + PDMS sensor material with the sandwich structure reported in our previous paper [33] was also prepared to supplement some data on the conversion of temperature to resistance. The difference between the two materials is that the conductive path of the PDMS + PDMS/GaInSn + PDMS sensor material is formed by the lap bonding of the GaInSn microdroplets dispersed in the PDMS matrix.

### 2.3. Characteristic of the PDMS/Capillary GaInSn Flexible Sensing Material

#### 2.3.1. Recording of Resistance Signals with Temperature Changes

To define the thermal sensitivity of the PDMS/capillary GaInSn flexible sensing material, the real-time evolution of the electrical signal-resistance changing (Δ*R*/*R*_0_) of the material with temperature change was recorded. The Δ*R*/*R*_0_ is the resistance change normalized by the initial resistance, which is an important parameter in flexible conductive materials research and is commonly used to measure the resistance change of flexible sensing materials [25,38,39].

The temperature change was realized by the temperature control programs of a muffle furnace (KSL-1200x, Hefei Kejing Material Technology Co., Ltd., Hefei, China). The Δ*R*/*R*_0_ changing with time during the temperature-rising processes were monitored by the bench-type digital multimeter (Keysight 34461A, Keysight Technology Co., Ltd., Santa Rosa, CA, USA) on a two-wire mode. The automatic mode was used with the measurement option set to Resistance 2 W. For comparison, the PDMS + PDMS/GaInSn + PDMS sensor material with the sandwich structure was also heated in the muffle furnace, while the Δ*R*/*R*_0_ changing with time was recorded.

#### 2.3.2. Recording of Resistance Signals with Cyclic Stretching, Cyclic Compression and Cyclic Bending Testing

To study the force sensitivity of the PDMS/capillary GaInSn flexible sensing material, the Δ*R*/*R*_0_ with several deformation modes (cyclic stretching, cyclic compressing and cyclic bending) of the flexible sensing material were measured. The cyclic stretching, cyclic compressing and cyclic bending were conducted on an electronic universal testing machine (UTM4104, Shenzhen Suns Technology Stock Co., Ltd., Shenzhen, China). The Δ*R*/*R*_0_ changing with time during the three kinds of deformation processes were also monitored by the Keysight 34461A on a two-wire mode at room temperature. 

The strain gauge factor (*GF* = (Δ*R*/*R*_0_)/*ε*_1_, *ε*_1_ is the tensile strain) [40,41] was used to represent the sensitivity of the sensing material during the stretching process. The compression ratio was used to measure the degree of compression during the cyclic compression process. The calculation formula for the compression ratio is shown in Equation (1):

(1) $$\varepsilon_{2}=\frac{a}{t}$$

where *ε*_2_ is the compression ratio; *a* is the relative displacement at the point of force application on the sample (mm); *t* is the average thickness of the sample (mm). The relative bending curvature was used to measure the degree of bending during the cyclic bending process. The calculation formulas for the relative bending curvature are shown in Equations (2) and (3):

(2) $$r=\frac{a^{2}+b^{2}}{2at}$$

(3) $$k=\frac{1}{r}=\frac{2at}{a^{2}+b^{2}}$$

where *r* is the relative bending radius; *k* is the relative bending curvature for three-point bending test; *b* is half the distance between the fixed ends of the sample (mm); *a* is also the relative displacement at the point of force application on the sample (mm); *t* is also the average thickness of the sample (mm). In this study, the maximum *ε*_2_ for the cyclic compression was 0.5, and the maximum *k* for the cyclic bending was 0.16.

#### 2.3.3. Recording of Resistance Signals Under Impact and Rolling Pressure

To define the impact and rolling pressure sensitivity of the material, five different weights (10 g, 20 g, 50 g, 100 g, 200 g) were used to free-fall from the same height, then impact the material, and roll over the material, while recording the resistances of the material.

#### 2.3.4. Testing of Biological Signal Transmission Capability

To detect the usability of the flexible sensing material as a biological sensor, the flexible sensing material was bound to human joints of fingers, wrists, elbows and the sole of shoes. Then repeat the joint bent-joint straight motion several times, which led to the resistance change of the flexible sensing material pasted on the joints. Also, repeat the stand up-crouch motion several times, which resulted in the resistance change of the flexible sensing material pasted on the sole of the shoes. The real-time resistance changes of the flexible sensing materials during the cyclic joint motion and the stand up-crouch motion were recorded by the Keysight 34461A.

## 3. Results and Discussion

### 3.1. Temperature Change Generate Electrical Signal

**Figure 2:**
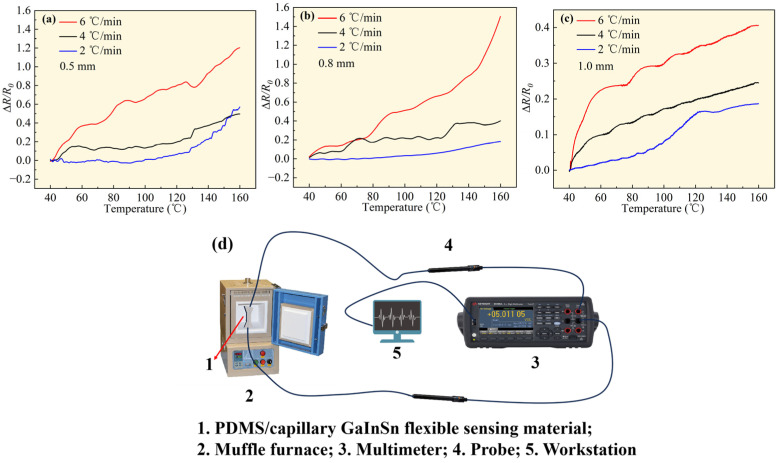
Δ*R/R*_0_ of the PDMS/capillary GaInSn flexible sensing material at different heating rates with the conductive path diameters of (**a**) 0.5 mm, (**b**) 0.8 mm, and (**c**) 1.0 mm. (**d**) Schematic diagram of the equipment connection for temperature sensitivity testing.

The silicone capillary filled with GaInSn served as the conductive pathway, so the diameter of the silicone capillary was defined as the diameter of the conductive pathway. The electrical performance test results of the PDMS/capillary GaInSn flexible sensing materials with conductive pathway diameters of 0.5 mm, 0.8 mm, and 1.0 mm under temperature changes are shown in Figure 2a, Figure 2b, and Figure 2c, respectively. Each data plot consists of three different curves representing real-time resistance changes at heating rates of 2 °C/min, 4 °C/min, and 6 °C/min. The schematic diagram of the equipment connection for the temperature sensitivity testing process is shown in Figure 2d.

The resistance of the PDMS/capillary GaInSn flexible sensing materials underwent regular and significant increases when temperature increased. First, the LM can be viewed as a mixture of the positive ionic fluid and the free electron gas. With the increase in temperature, the ions and electrons in the LM were excited by heat, which made them vibrate more, thus reducing the mobility of the electrons and leading to a decrease in electrical conductivity. Moreover, the outer layer PDMS was expanded by heat, which squeezed the LM conductive channel, reduced the cross-sectional area of the conductive channel, and made the resistance increase. Moreover, with a higher heating rate, the above two effects were more pronounced, resulting in poorer conductivity and a faster increase in resistance. This enables the sensing material to transform the temperature signal that is not convenient for real-time recording into the electrical signal that is convenient for real-time recording.

**Figure 3:**
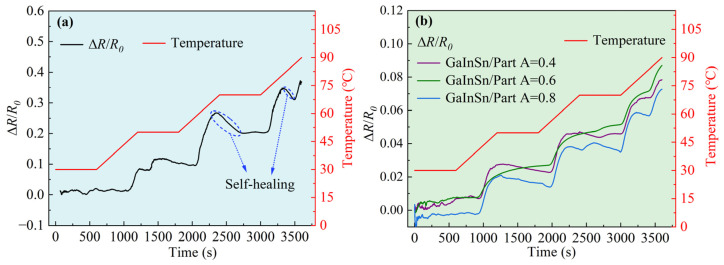
Δ*R/R*_0_ of (**a**) the PDMS/capillary LM flexible sensing material and (**b**) the PDMS + PDMS/GaInSn + PDMS sensor materials under a specific heating procedure.

In addition, Figure 3a represents the resistance change (black line) of the PDMS/capillary GaInSn flexible sensing material under a specific heating program (red line): it was kept warm at 30 °C for 10 min, followed by a heating rate of 2 °C/min to reach 50 °C, and according to the illustrated heating curve, insulation and heating operations were repeated until the temperature reached 90 °C. Figure 3a indicated that the resistance increased with temperature, which was consistent with the results above. However, when stopping heating and insulation for a period of time, the resistance first exhibited a decrease and then stayed constant. This was due to the PDMS/capillary GaInSn flexible conductive composite material possessing “self-repairing” properties to a certain extent. The flowing LM GaInSn served as the repair material, re-establishing the pathways at weak contact points, thus restoring the sample’s conductivity. This suggested that the material might still achieve a good conductivity effect through self-repair, even in harsh conditions such as high temperatures. 

Under the same heating procedure, the resistance with temperature of the PDMS + PDMS/GaInSn + PDMS sensor materials with different GaInSn contents is shown in Figure 3b. The overall trend of the resistance and the temperature was the same, both of which were step-like. When the temperature was constant, the resistance was also basically constant. When the temperature increased, the resistance also increased. The reason was that the thermal expansion coefficient of the PDMS was larger than that of the GaInSn microdroplets. Therefore, when the sample was heated, the distance between the GaInSn microdroplets was widened, and the conductive network was stretched to increase the resistivity. Since the conductive path of the PDMS + PDMS/GaInSn + PDMS sensor material was formed by the lap of LM microdroplets dispersed in the PDMS matrix, the fluidity of the LM was weaker than that of the PDMS/capillary GaInSn material, and the self-repair ability of the PDMS + PDMS/GaInSn + PDMS material was weaker than that of the PDMS/capillary GaInSn material. 

### 3.2. Cyclic Stretching, Cyclic Compression and Cyclic Bending Generates Electrical Signal

**Figure 4:**
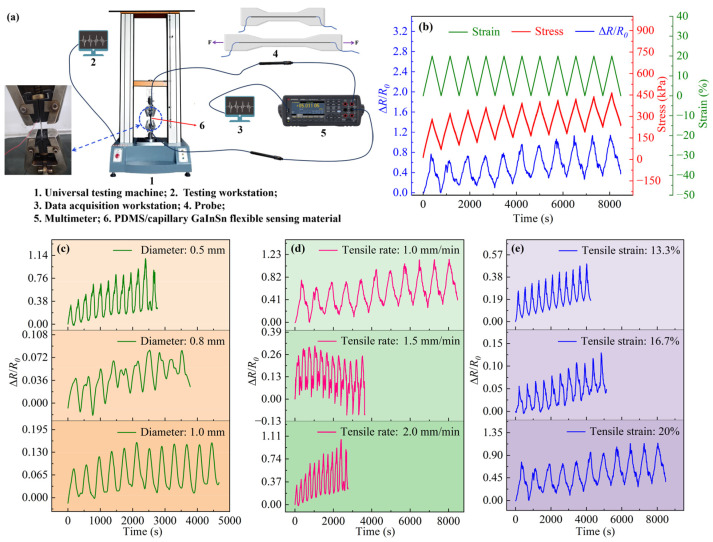
(**a**) Diagram of the stretching equipment connection. (**b**) Correspondence of stress, strain, and Δ*R*/*R*_0_ under the cyclic stretching operations. The Δ*R*/*R*_0_ evolutions of the GaInSn flexible sensing material samples under cyclic stretching operations: (**c**) tensile strain 20%, tensile rate 2.0 mm/min, diameters 0.5/0.8/1.0 mm, (**d**) tensile strain 20%, diameter 0.5 mm, tensile rates of 1.0/1.5/2.0 mm/min, and (**e**) tensile rate 1.0 mm/min, diameter 0.5 mm, tensile strains 13.3%, 16.7%, and 20%.

The PDMS/capillary GaInSn flexible sensing material samples exhibited good tensile mechanical properties with a fracture elongation of 127%. The electrical signal Δ*R*/*R*_0_ was monitored when the samples were stretched repeatedly via the electronic universal testing machine, as shown in Figure 4a. The stress–strain and the Δ*R*/*R*_0_ change are shown in Figure 4b. The resistance of the material increases with the strain as the elongation increases. This was primarily due to the elongation of the conductive path and the reduction in the cross-sectional area during the stretching process. Since the resistance was inversely proportional to the cross-sectional area and directly proportional to the length of the conductive path, the resistance of the material increased with the increase in strain. Therefore, it can be observed that under small strains, the resistance of the “capillary structure” PDMS/capillary GaInSn flexible sensing material changed with strain, thereby converting mechanical tensile strain signals into electrical signals.

Figure 4c shows the Δ*R*/*R*_0_ versus time under cyclic stretching testing with a maximum tensile strain of 20%, a tensile rate of 2.0 mm/min, and diameters of 0.5 mm, 0.8 mm, and 1.0 mm for the PDMS/capillary GaInSn flexible sensing materials. The Δ*R*/*R*_0_ of the samples with different conductive pathway diameters all changed periodically with the strain. The maximum and the minimum values of the Δ*R*/*R*_0_ for each cyclic stretch tended to become higher as the cyclic stretch went on, indicating that the conductive path was slightly damaged by the deformation, resulting in increases in the resistance. A sample with a diameter of 0.5 mm was found to have a more stable electrical signal during the cyclic stretching and have the highest sensitivity (average *GF* = 0.724) among each sample. Therefore, a sample with a diameter of 0.5 mm was chosen as the research object in the following tests.

Figure 4d illustrates the Δ*R*/*R*_0_ versus time under cyclic stretching testing at a maximum tensile strain of 20% for the 0.5 mm diameter PDMS/capillary GaInSn flexible sensing material at tensile rates of 1.0 mm/min, 1.5 mm/min, and 2.0 mm/min. The Δ*R*/*R*_0_ changed relatively steadily and periodically with the tensile strain with tensile rates from 1.0 mm/min to 2.0 mm/min.

Figure 4e shows the Δ*R*/*R*_0_ versus time under cyclic stretching testing with different maximum tensile strain of 13.3%, 16.7%, and 20% at a tensile rate of 1.0 mm/min for the 0.5 mm diameter PDMS/capillary GaInSn flexible sensing material. The samples were found to have a relatively stable electrical signal during cyclic stretching. The Δ*R*/*R*_0_ of the sample changed periodically with the tensile strain when the tensile strains were 13.3% to 20%. The maximum value of *GF* was 0.912, with a tensile rate of 1.0 mm/min and a tensile strain of 20%, indicating that the sensing material had the highest sensitivity under this condition. The maximum *GF* of the capillary structure material was higher than that of the PDMS + PDMS/GaInSn + PDMS sensor material with the lap bonding of LM microdroplets dispersed in the PDMS matrix as the conductive path [32].

In conclusion, in the cyclic stretching operation of the PDMS/capillary GaInSn flexible sensing materials, the Δ*R*/*R*_0_ could change periodically and stably with the strain, when the conductive pathway diameters were 0.5–1.0 mm, the cyclic tensile strain was less than 20%, and the tensile rate was no more than 2.0 mm/min.

In addition to the cyclic stretching being able to produce an electrical signal, the influence of other deformation methods, such as cyclic compression and cyclic bending were also studied. 

**Figure 5:**
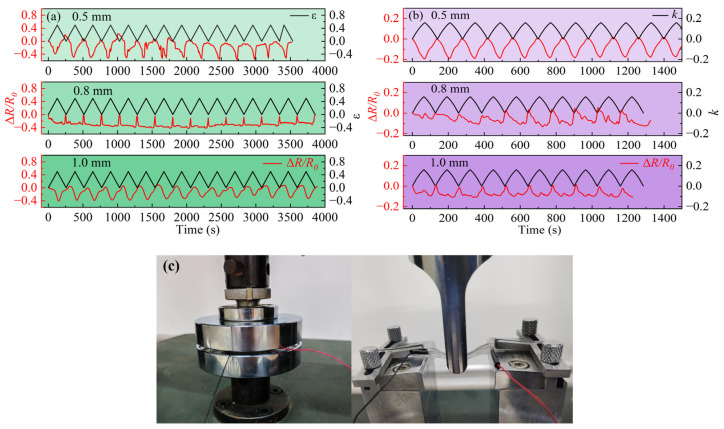
Δ*R*/*R*_0_ of the PDMS/capillary GaInSn sensing materials with different conductive path diameters of 0.5 mm, 0.8 mm, and 1.0 mm during (**a**) the compression process and (**b**) the bending process. (**c**) Actual operation pictures of the compression and bending.

The Δ*R*/*R*_0_ evolution of the PDMS/capillary GaInSn flexible sensing materials with conductive pathway diameters of 0.5 mm, 0.8 mm, and 1.0 mm are shown in Figure 5a under the cyclic compression operations with a maximum *ε*_2_ of 0.5. The Δ*R*/*R*_0_ of the samples changed periodically with the *ε*_2_, on the whole, and the resistance of the sensing material was inversely proportional to the *ε*_2_. That was, to some extent, as the *ε*_2_ increased, the conductivity of the material improved. The Poisson ratio of rubber is 0.5, the compression resulted in smaller sample thickness and larger width and length, so the cross-sectional area of the capillary decreased and the length became larger, while the LM in the tube generated axial convection, which enhanced the ion and free electron mobility in the LM. The reduction in the cross-sectional area and the increase in the length led to an increase in the resistance, while the axial convection of the LM reduced the resistance. However, due to the changes in cross-sectional area and length being so weak, the influence of axial convection dominated and eventually, the compression led to a smaller resistance.

The Δ*R*/*R*_0_ evolution of the PDMS/capillary GaInSn flexible sensing materials with conductive pathway diameters of 0.5 mm, 0.8 mm, and 1.0 mm are shown in Figure 5b under the cyclic bending operations with a maximum *k* of 0.16. The Δ*R*/*R*_0_ of the samples also changed periodically with the *k*, on the whole, the resistance of the sensing materials was inversely proportional to the *k*. That is, to some extent, as the bending degree increased, the conductivity of the material increased. This was because, under bending conditions, the GaInSn in the conductive path was subjected to external forces, flowing in the direction with the maximum radial force and reducing potential energy. With the increase in the flowing capability of the GaInSn, the mobility of the free electron clouds increased, the ability to conduct current increased, and thus the resistance of the material decreased. The actual operation pictures of compression and bending are shown in Figure 5c.

In conclusion, Figure 5 demonstrates that the PDMS/capillary GaInSn flexible sensing materials possess the capability to convert and transmit compression stress and bending strain signals into electrical signals under specific conditions.

**Figure 6:**
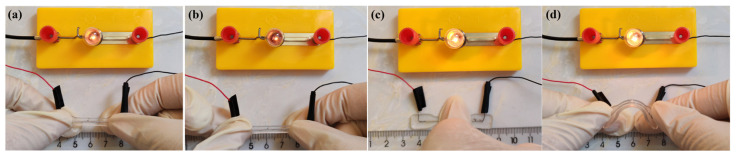
The brightness comparison of the bulb under the four conditions of the material in the (**a**) original, (**b**) stretched, (**c**) compressed, and (**d**) bent states.

As shown in Figure 6, the brightness of the bulbs in the original, stretched, compressed, and bent states was consistent with the change trends of the resistance above, and the smaller the resistance, the brighter the bulb.

### 3.3. Impact and Rolling Generates Electrical Signal

**Figure 7:**
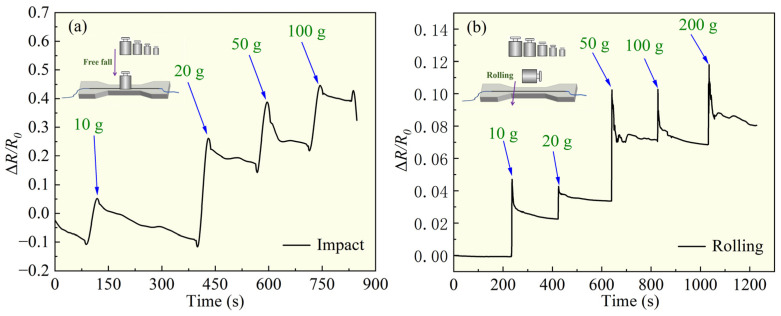
Δ*R/R*_0_ of the material under (**a**) impact operations and (**b**) rolling operations.

As shown in Figure 7, the PDMS/capillary GaInSn flexible sensing materials were tested under different intensities of impact or rolling pressure. The two small schemes on the upper left corner of the two figures displayed the impact and rolling measurement progress. The materials exhibited varying electrical signals under different levels of force. In Figure 7a, the resistance of the sample increased after impact and the increment of the resistance is proportionate to the weight of the impact weights. In Figure 7b, the resistance of the sample increases after rolling, and the magnitude of the resistance increase is also proportionate to the weight of the rolling weights. This was because, during the impact or rolling, the sample experienced radial compression due to external forces, leading to a sudden decrease in the diameter of the conductive path and an increase in resistance. Additionally, the flow capability of the LM GaInSn was restricted, hindering the movement of free electrons and resulting in an increase in sample resistance. 

This indicated that under certain impact or rolling conditions, the PDMS/capillary GaInSn flexible sensing materials could also exhibit changes in resistance signals, meaning that the material could convert impact pressure and rolling action signals into electrical signals, and it also had the ability to transmit these signals.

### 3.4. Analysis of Biological Signal

**Figure 8:**
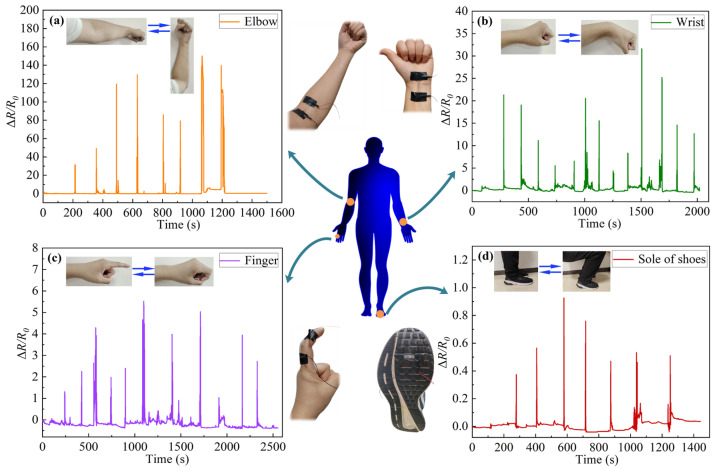
Movement signals collection of the (**a**) elbow, (**b**) wrist, (**c**) finger and (**d**) the sole of foot.

Based on the above research results, in order to determine the feasibility of the PDMS/capillary GaInSn flexible sensing material to monitor body movement signals, the material was pasted outside the finger joints, and inside the wrist joints and elbow joints and the sole of the shoes. The resistance changes of the material during the joint activity or stand up-crouch motion were recorded as shown in Figure 8. It can be seen from Figure 8a–c that the joint bending caused the resistance to become larger, and the joint recovery restored the resistance. It can be seen from Figure 8d that when squatting, the resistance increased, and when standing up, the resistance recovered. This demonstrated its potential application as a flexible biosensor. 

The Δ*R*/*R*_0_ was much higher in the joint cases than in the cases of cyclic stretching, cyclic compression and cyclic bending performed by the tensile testing machine. The difference in the stresses applied was one reason, and the stress in joint cases was much higher than that in the machine cases. Another important reason was that the mechanism of the two cases was different; for example, in the finger joint case, the sensing material was stretched plus bent for it was pasted outside the joint. In the wrist and elbow joint cases, the sensing material was compressed plus bent for it was pasted inside the joint. Also, the bending in the joint cases was not simple three-point bending. As a result, the flow of LM in the capillary was complicated. Therefore, the Δ*R*/*R*_0_ was much higher in the joint cases than in the testing machine cases. The joint motion pattern was difficult to simulate with the simple stretching, compression and three-point bending by the tensile testing machine. 

Therefore, the PDMS/capillary GaInSn flexible sensing material can convert these biomechanical limb motion signals into electrical signals. By making the PDMS/capillary GaInSn flexible sensing material thinner and finer, it may respond to tiny strains, since the human skin produces tiny strains due to breathing, heart beating and pulse beating. On the other hand, the PDMS/capillary GaInSn flexible sensing material is sensitive to temperature changes, and the body temperature is an important reflection of the body’s health status, and the material can also convert the body temperature into electrical signals. Therefore, the PDMS/capillary GaInSn flexible sensing material has a good application prospect in real-time monitoring of human limb motion and health information in the biomedical field. 

## 4. Conclusions

A flexible sensing material with high force and thermal sensitivity was successfully prepared utilizing GaInSn with good conductivity and flexible silicone capillary to construct signal network channels, and a PDMS encapsulation layer for shaping and providing tensile properties. The electrical performance analysis results confirmed that the resistance of the sensing materials was responsive to the temperature, with increasing temperature to a certain extent, the real-time resistance of the flexible sensing material increased, indicating its ability to transmit temperature signals. The resistance of the material was also sensitive to external forces, such as cyclic stretching, cyclic compression, cyclic bending, impact and rolling. The Δ*R*/*R*_0_ changed periodically and stably with the cyclic operation when the conductive pathway diameter was 0.5–1.0 mm, the cyclic tensile strain was less than 20%, and the cyclic tensile rate was no more than 2.0 mm/min in the cyclic stretching processes, the *ε*_2_ was no more than 0.5 in the cyclic compression processes, and the *k* was no more than 0.16 in the cyclic bending processes. Moreover, the material exhibited sensitivity in detecting biological signals, such as the movement of finger joints, wrist joints, elbow joints and the stand up-crouch motion. In conclusion, this work provides a method for preparing a sensing material with the capillary structure, which was confirmed to be sensitive to force and heat, and it produced different types of *R* signals under different deformations and different temperatures, based on which it could accurately judge the different external environment changes. Therefore, the material can be used as a deformation and temperature sensor in the field of flexible wearable and robotic applications. 

## Data Availability

All data are contained within the article.
